# Trait Mindfulness and Functional Connectivity in Cognitive and Attentional Resting State Networks

**DOI:** 10.3389/fnhum.2019.00112

**Published:** 2019-04-12

**Authors:** Tracie D. Parkinson, Jennifer Kornelsen, Stephen D. Smith

**Affiliations:** ^1^Department of Psychology, University of Manitoba, Winnipeg, MB, Canada; ^2^Department of Radiology, University of Manitoba, Winnipeg, MB, Canada; ^3^Department of Psychology, University of Winnipeg, Winnipeg, MB, Canada

**Keywords:** functional connectivity, resting state networks, trait mindfulness, default mode network, salience network, central executive network, dorsal attention network, ventral attention network

## Abstract

Mindfulness has been described as an orienting of attention to the present moment, with openness and compassion. Individuals displaying high *trait* mindfulness exhibit this tendency as a more permanent personality attribute. Given the numerous physical and mental health benefits associated with mindfulness, there is a great interest in understanding the neural substrates of this trait. The purpose of the current research was to examine how individual differences in trait mindfulness associated with functional connectivity in five resting-state networks related to cognition and attention: the default mode network (DMN), the salience network (SN), the central executive network (CEN), and the dorsal and ventral attention networks (DAN and VAN). Twenty-eight undergraduate participants completed the Five-Facet Mindfulness Questionnaire (FFMQ), a self-report measure of trait mindfulness which also provides scores on five of its sub-categories (*Observing*, *Describing*, *Acting with Awareness*, *Non-judging of Inner Experience*, and *Non-reactivity to Inner Experience*). Participants then underwent a structural MRI scan and a 7-min resting state functional MRI scan. Resting-state data were analyzed using independent-component analyses. An analysis of covariance (ANCOVA) was performed to determine the relationship between each resting state network and each FFMQ score. These analyses indicated that: (1) trait mindfulness and its facets showed increased functional connectivity with neural regions related to attentional control, interoception, and executive function; and (2) trait mindfulness and its facets showed decreased functional connectivity with neural regions related to self-referential processing and mind wandering. These patterns of functional connectivity are consistent with some of the benefits of mindfulness—enhanced attention, self-regulation, and focus on present experience. This study provides support for the notion that non-judgmental attention to the present moment facilitates the integration of regions in neural networks that are related to cognition, attention, and sensation.

## Introduction

Originating in Buddhist traditions, mindfulness training has made its way into Western culture as a method to reduce stress, enhance emotional regulation, and reduce symptoms in a variety of mental health disorders (Teasdale et al., 1995; Farb et al., 2012; Cavanagh et al., 2013; Tabak et al., 2015). Indeed, mindfulness has been shown to reduce symptoms of depression (Chiesa et al., 2015), bipolar disorder (Ives-Deliperi et al., 2013), anxiety (Cavanagh et al., 2013), chronic dysphoria (Farb et al., 2012), and borderline personality disorder (O’Connell and Dowling, 2014), in addition to increasing self-esteem (Bajaj et al., 2016), reducing substance cravings (Witkiewitz et al., 2013), and enhancing overall quality of life (Chiesa et al., 2015). With such extensive benefits in clinical and non-clinical populations, there has been a growing interest in understanding mindfulness. While specific techniques used to treat these conditions may vary, mindfulness practices, in general, involve paying attention to the moment, purposefully and non-judgmentally (Kabat-Zinn, 1994) with an attitude of openness, compassion, and acceptance to the experience (Brown and Ryan, 2003; Bishop et al., 2004; Shapiro et al., 2006; Baer, 2011). This process typically leads to a perspective shift in which an individual detaches the contents of their thoughts and feelings from the self and instead engages in a clear and objective observation of his or her moment-to-moment experience (Shapiro et al., 2006). Given its numerous physical and psychological benefits, it is not surprising that a substantial number of studies have investigated the mechanisms underlying mindfulness (Ives-Deliperi et al., 2011; Farb et al., 2012; Dickenson et al., 2013; Gotnik et al., 2016; Haase et al., 2016; Kral et al., 2018).

The majority of the empirical investigations of mindfulness have focused on interventions that allow an individual to intentionally incorporate mindful practices into their daily lives (i.e., *state* mindfulness). However, personality researchers have noted that individuals also vary in their natural tendency to adopt a mindful perspective across experiences and contexts (i.e., *trait* mindfulness; Lu et al., 2014; Doll et al., 2015; Kong et al., 2016; Bilevicius et al., 2018).

Previous studies have found that state and trait mindfulness are very similar for experienced meditators; the long-term practice of entering and maintaining a mindful state appears to transform that transient mindful perspective of state mindfulness into a more stable, trait-like characteristic (Hölzel et al., 2011; Tanay and Bernstein, 2013; Wheeler et al., 2017). In contrast, state and trait mindfulness appear to be relatively independent processes in individuals who are meditation-naïve, with different personality characteristics and facets of mindfulness involved in each process (Thompson and Waltz, 2007). In the current research, we will assess brain activity in meditation-naïve participants. Specifically, we will use functional magnetic resonance imaging (fMRI) to examine how different facets of trait mindfulness are related to the functional connectivity of resting-state networks in the brain. This strategy will allow us to examine the neural underpinnings of trait mindfulness without the potential confound of mindfulness meditation training, which would blur the line between state and trait mindfulness.

Most previous fMRI investigations of trait mindfulness have examined how neural activity during a behavioral task differs between individuals who score high or low on a measure of this construct. For example, Dickenson et al. (2013) observed that during a focused breathing task, people who scored higher on a measure of trait mindfulness show greater activity in the temporo-parietal junction, superior parietal lobule, and dorsolateral prefrontal cortex (DLPFC) than did people who scored low on this measure. These brain areas are involved with orienting and sustaining of attention, a result consistent with cognitive psychology studies showing that mindfulness is associated with superior attentional abilities (Quaglia et al., 2015; Quan et al., 2018). Creswell et al. (2007) compared levels of trait mindfulness to neural activation patterns during an affect labeling task. They reported that during labeling, trait mindfulness was associated with a reduced amygdala response and more widespread prefrontal cortex (PFC) activation, highlighting particularly heightened activity in the medial PFC (MPFC). These researchers interpreted their findings to suggest that during affect labeling, greater trait mindfulness is linked to cortical regulation of limbic responses.

A component of trait mindfulness—the tendency to observe—has also been shown to predict activation of neural regions when attending to one’s emotions (Frewen et al., 2010). Observing entails attending to your thoughts, feelings, or emotions without judgment (Baer et al., 2006). Individuals who tended to engage in mindful observation showed larger activity in the dorsomedial PFC (DMPFC) while individuals listened to both negative and positive audio emotional vignettes than did participants who scored low on this measure (Frewen et al., 2010). The authors suggested that this activation provides evidence that mindfulness involves an internal emotional reflection process that recruits the DMPFC (Frewen et al., 2010).

Voxel-based morphometry research has identified a positive correlation between trait mindfulness and gray matter volume in the bilateral anterior cingulate cortex (ACC) and a negative correlation with the gray matter volume of the left orbitofrontal cortex (Lu et al., 2014). This result is consistent with the role of the ACC in both attentional control and emotional regulation (Mohanty et al., 2007; Giuliani et al., 2011; Stevens et al., 2011).

An additional neuroimaging technique used to investigate the neural underpinnings of trait mindfulness is resting-state fMRI. In these studies, neural activity is measured while participants lie awake in the scanner but are not performing a cognitive or perceptual task (i.e., they are “at rest”; for a review, see Raichle, 2015). Although no task is being performed, spontaneous neuronal activity throughout the brain continues to occur. Importantly, the neuronal activity is not random; instead, the activity of groups of structurally disparate brain areas frequently correlate, suggesting that these areas function as a network (Raichle et al., 2001; Damoiseaux and Greicius, 2009). Researchers have identified a number of different resting-state networks in the brain and have examined how the magnitude of the correlation of activity in different regions, known as functional connectivity, is related to different cognitive abilities and clinical conditions (e.g., Raichle et al., 2001; Damoiseaux et al., 2006; Broyd et al., 2009; Damoiseaux and Greicius, 2009; van den Heuvel and Hulshoff Pol, 2010; Rosazza and Minati, 2011).

The functional connectivity of three of the most commonly studied networks—the default mode network (DMN), salience network (SN), and central executive network (CEN)—are involved with cognitive, attentional, and emotional processes that are related to both state and trait mindfulness (Hasenkamp et al., 2012; Taylor et al., 2013; Doll et al., 2015; Bilevicius et al., 2018). The DMN is comprised of nodes in the precuneus, posterior cingulate cortex, MPFC, lateral anterior temporal lobe, and posterior parietal lobe. This network shows increased activity during mind-wandering, self-referential thought, remembering the past, and thinking about the future (Andrews-Hanna, 2012). Several of these functions are, in many ways, antithetical to mindfulness. The SN consists of the insula and anterior cingulate gyrus. It is involved with orienting an individual’s attention to external and internal events based on sensory and limbic inputs while also mediating functions between the other networks (Seeley et al., 2007; Bressler and Menon, 2010; Bonnelle et al., 2012). For these functions, the SN would require essential components of mindfulness, present moment attention, and bodily self-awareness. The CEN consists of nodes in the DLPFC, the ACC/DMPFC, and the posterior parietal cortex. It is involved with several executive processes including attentional control, memory, language, and visual processes (Bressler and Menon, 2010; Rosazza and Minati, 2011). Attentional control is a requirement of focusing during a mindfulness practice; thus, the CEN also has functions related to mindfulness.

Two additional resting-state networks that have received considerably less attention from mindfulness researchers are the dorsal and ventral attention networks (DAN and VAN, respectively). The DAN and VAN are both attentional frontoparietal networks that operate during sensory orientation processes (Fox et al., 2006). The DAN consists of the lateral PFC, posterior inferior parietal cortex, and intraparietal cortex (Corbetta et al., 2008). The DAN is involved with voluntarily orienting and maintaining attention to a location (Corbetta et al., 2000) and is considered to be a goal-driven attentional network that uses internal goals or expectations to attend to sensory stimuli (Corbetta et al., 2008). Mindfulness requires sustained, focused attention; thus, the functions of the DAN are directly linked to mindfulness. The VAN contains nodes in the temporo-parietal junction, ACC, and anterior insula. It is another attentional network that is also involved with the detection of salient environmental information; however, this network is stimulus-driven and is implicated in detecting unexpected information (Corbetta et al., 2008). Unlike the SN, the VAN does not rely on interoception to orient attention to salient information in the environment. Mindfulness requires attention to the external environment, a primary function of the VAN. Understanding how these five cognitive and attentional networks relate to trait mindfulness would provide a valuable, comprehensive addition to a discussion of the neural correlates of trait mindfulness.

An additional advantage of the current research is that it utilizes a more nuanced measure of mindfulness than was used in previous studies. For example, Bilevicius et al. (2018) analyzed the relationship between trait mindfulness and functional connectivity in four resting state networks, the DMN, SN, CEN, and DAN. In their study, trait mindfulness was measured using the Mindful Attention Awareness Scale (MAAS), a self-report measure that assesses the attentional aspect of trait mindfulness (Brown and Ryan, 2003). Trait mindfulness negatively correlated with the left medial frontal gyrus, a mind-wandering region of the DMN, whereas a positive relationship was reported in the SN with the left ACC, a region associated with attentional control (Bilevicius et al., 2018). Doll et al. (2015), on the other hand, measured trait mindfulness with both the MAAS and the Freiburg Mindfulness Inventory (FMI) in participants who completed a brief mindfulness intervention (Doll et al., 2015). They found that inter-network functional connectivity between the DMN and SN and between the SN and left CEN positively correlated with mindfulness scores (Doll et al., 2015). Although these results were both novel and informative, both the MAAS and FMI are unidimensional assessments of trait mindfulness (Walach et al., 2006).

The current study used the Five-Facet Mindfulness Questionnaire (FFMQ), a scale created by Baer et al. (2006) using a composite of the Southampton Mindfulness Questionnaire, Cognitive and Affective Mindfulness Scale-Revised, Kentucky Inventory of Mindfulness Skills, MAAS, and FMI. On the FFMQ, participants rate their responses to each item on a 5-point Likert scale from 1 (“never or very rarely true”) to 5 (“very often or always true”). Statements cover five sub-areas pertaining to mindfulness, including *Observing*, *Describing*, *Acting with Awareness, Non-Judgment to Inner Experience*, and *Non-Reactivity to Inner Experience* (Baer et al., 2006). *Observing* refers to the observation of internal and external stimuli (e.g., “When I’m walking, I deliberately notice the sensations of my body moving”). *Describing* includes statements assessing a person’s ability to express their experiences, thoughts, and emotions (e.g., “I can easily put my beliefs, opinions, and expectations into words”). *Acting with awareness* (henceforth *Acting*) includes statements that refer to paying attention in the present moment (e.g., “When I do things, my mind wanders off and I’m easily distracted”). *Non-judgment to inner experience* (henceforth *Non-Judging*) statements assess the degree to which an individual rates their thoughts, feelings, and emotions as good or bad (e.g., “I make judgments about whether my thoughts are good or bad”). Finally, the *Non-reactivity to inner experience* (henceforth *Non-Reactivity*) items assess the degree to which an individual reacts to their feelings, emotions, and thoughts (e.g., “I perceive my feelings and emotions without having to react to them”). Psychometric studies of the FFMQ have shown that it possesses consistently good reliability and validity across a variety of cultures (i.e., Baer et al., 2008; Deng et al., 2011; Heeren et al., 2011; Cebolla et al., 2012; de Bruin et al., 2012; Giovannini et al., 2014; Taylor and Millear, 2016) with a five-factor hierarchical structure for meditators and a four-factor hierarchical structure (without the *Observing* facet) for non-meditators and clinical samples (Williams et al., 2014; Aguado et al., 2015). Because the present study used meditation naïve participants, the *Observing* facet will be interpreted with caution.

In the current research, overall trait mindfulness scores (FFMQ_Total_) and values for each of the five facets of mindfulness were entered as covariates into analyses of functional connectivity. These ANCOVAs allowed us to examine whether individual differences in self-reported trait mindfulness were related to differences in the functional connectivity of five cognitive and attentional resting state networks (the DMN, SN, CEN, DAN, and VAN). Functional connectivity in each resting state network was identified using independent component analysis (ICA). This approach is data-driven, without restriction to* a priori* regions-of-interest. This allowed us to identify novel and/or counterintuitive results not previously reported in the literature.

## Materials and Methods

### Participants

Twenty-nine meditation-naïve undergraduate students (15 females, age (M ± SD) = 19.89 ± 2.74, range = 18–29 years) from the University of Winnipeg volunteered to participate. Exclusion criteria included participants with a history of psychiatric or neurological disorders, metal in the body, pregnancy, or claustrophobia. Participants provided written consent and completed magnetic resonance safety screening prior to entering the MRI scanner. The University of Winnipeg Human Research Ethics Board and the Bannatyne Human Research Ethics Board provided ethical approval for this study. Participants received a $50 honorarium for their participation.

### Psychological Measure

All participants completed the FFMQ prior to or after entering the scanner. The overall score and sub-scores of the FFMQ were tallied using standardized scoring guidelines (Baer et al., 2006). Scores included the overall score on the FFMQ (FFMQ_Total_) and the five subscales or facets of mindfulness (*Observing, Describing, Acting, Non-Judging*, and* Non-Reactivity*). A high overall score on the FFMQ indicates elevated trait mindfulness. Higher scores in any of the five individual components indicate greater expression of that facet of mindfulness.

### Data Acquisition

Structural and functional MRI data were acquired for all participants using a 3T Siemens TRIO MRI scanner (Siemens, Erlangen, Germany). Following the initial localizer scan, a 3D high-resolution anatomical MRI was acquired. This high resolution T1-weighted gradient-echo scan was 8 min in duration and was performed using an MP-RAGE sequence. This scanning sequence utilized the following parameters: 1-mm slice thickness, 0 mm gap, TR/TE = 1,900/2.2 ms, in plane resolution 0.94 × 0.94 mm, 256 × 256 matrix, field of view (FOV) 24 cm.

Following the acquisition of structural images, a 7-min resting state functional MRI scan was performed. Resting state data was acquired with a whole brain echo planar imaging (EPI) sequence using the following scanning parameters: 140 volumes were obtained using 3-mm slice thickness, 0 gap, TR/TE = 3,000/30 ms, flip angle = 90°, 64 × 64 matrix, FOV 24 cm. Participants were instructed to close their eyes without falling asleep throughout the scanning session.

### Data Analysis

BrainVoyager QX 2.8 software (Brain Innovation, BV, Maastricht, Netherlands) was used to process imaging data and to perform all statistical analyses. Functional data were initially pre-processed using a trilinear/sync interpolation 3D motion correction, which examines movement output in six directions (three translations and three rotations). Visual inspection of participant movement was conducted. Data were not used if participants moved 2 mm or more in any of the six directions; however, none of the participants exceeded this threshold. After this initial step, further pre-processing was performed using a slice scan correction, high pass temporal filtering, and spatial smoothing (using an 8 mm full-width half-maximum Gaussian filter).

Following pre-processing, the functional data were co-registered to the high-resolution anatomical data. The anatomical data were spatially normalized to a standardized Talairach space and the functional time series data were transformed.

Single-subject ICA was performed for each individual using the fast ICA algorithm (Hyvärinen and Oja, 2000). Twenty independent components (ICs) were extracted from the data for all participants. A group-level ICA was then performed using a self-organizing group ICA (Sog-ICA) plugin. For this step, the most similar ICs for all participants were clustered at the group level, resulting in a total of 20 ICs. The 20 components were inspected manually and compared to the literature in order to identify each resting state network. Four cognitive/attentional resting state networks were identified (in separate ICs), including the DMN, SN, and CEN. While the CEN sometimes appears as two separate components as a right and left CEN (rCEN and lCEN; Damoiseaux et al., 2006; van den Heuvel et al., 2008; van den Heuvel and Hulshoff Pol, 2010), it appeared in the same component in this study. The fourth network was a frontoparietal network encompassing both the DAN and VAN, and will be henceforth referred to as the attentional network (ATN).

To determine the relationship between each resting state network and FFMQ scores, an analysis of covariance (ANCOVA) was performed. One ANCOVA was performed for every network using each facet of mindfulness as a covariate, with a predetermined significance level of *p* < 0.01. To illustrate, the component featuring the DMN was identified, and an ANCOVA was performed using the participants’ FFMQ_Total_ score as a covariate. Similar analyses were performed using the *Observing, Describing, Acting, Non-Judging*, and* Non-Reactivity* scores as covariates in separate ANCOVAs.

Each analysis produced cluster maps illustrating how scores on each of the six covariates associated with functional connectivity of a specific network. Voxels in these cluster maps exceeding a threshold (*p* < 0.01) represent areas whose functional connectivity in that network varied as a function of the covariate’s value. These maps were corrected for multiple comparisons using a Monte Carlo cluster threshold estimator correction plugin with 1,000 iterations, evaluated at *p* ≤ 0.01. Last, these maps were converted to volumes of interest (VOIs) to provide the coordinates of the peak intensity voxel, the number of significant voxels, and the probability value of the observed clusters.

VOI data were entered into Talairach Client software (Research Imaging Institute, Version 2.4.3, 2003–2015), providing an output on the anatomical label of the peak coordinate of each cluster including region, gyrus, hemisphere, and the specific Brodmann areas (BAs), when applicable. Each cluster was analyzed individually, using each cluster’s peak intensity coordinate and individual voxel information. Each cluster was reported with the corresponding correlation coefficient to indicate the strength and direction (positive or negative) of the relationship between functional connectivity for every network cluster and each facet of mindfulness. Additional tables consisting of the Montreal Neurological Institute (MNI) coordinates of each cluster are provided as Supplementary Material.

## Results

Data from one participant (female, age 24) was not included in any analyses due to electronic file corruption. Results refer to the remaining 28 participants. Statistics for FFMQ scores are featured in Table 1 below. The six scores for each participant were used as covariates in each ANCOVA with each resting state network.

**Table 1:** Scoring statistics and Cronbach’s Alpha (α) for each scale of the Five-Facet Mindfulness Questionnaire (FFMQ).

Scale	Mean	SD	Range	*α*
FFMQ_Total_	128.11	13.93	95–148	0.69
*Observing*	28.50	3.69	23–35	0.88
*Describing*	27.00	5.62	17–39	0.88
*Acting*	25.68	5.56	14–38	0.90
*Non-Judging*	24.86	6.89	13–38	0.90
*Non-Reactivity*	22.07	3.59	13–29	0.89

Internal reliability for trait mindfulness as represented by the overall FFMQ score was moderate and reliability for all of the FFMQ scales was high (Table 1). These results further attest to the fact that the FFMQ is a reliable measure of trait mindfulness.

The reporting of the functional connectivity data will be separated into four subsections, one for each network of interest. The Talairach coordinates for all network analyses indicate regions that showed an increased or decreased degree of functional connectivity as a function of FFMQ scores. Within each component, some of the clusters included traditional nodes from the resting state networks, whereas other clusters included non-traditional regions in individuals who scored high or low on FFMQ covariates. Orange voxels indicate that the FFMQ covariate values and the functional connectivity of that cluster are positively correlated, whereas blue voxels indicate that the FFMQ covariate values and the functional connectivity of that voxel are negatively correlated.

### Default Mode Network Functional Connectivity

**Figure 1:**
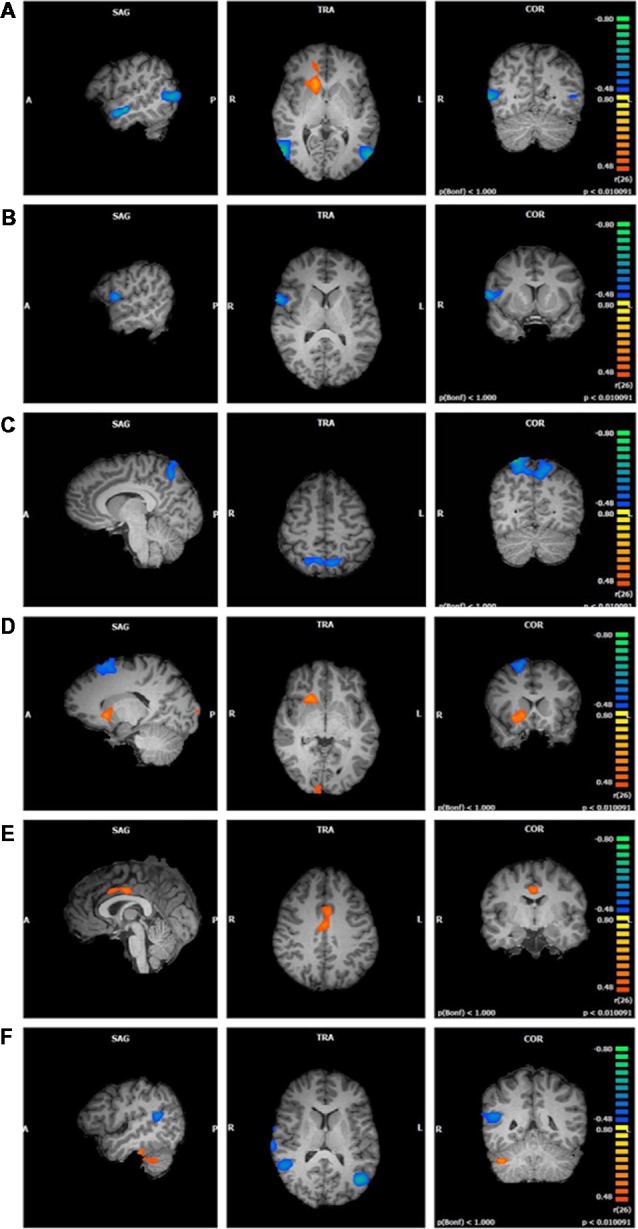
Functional connectivity of default mode network (DMN) clusters correlated with trait mindfulness and five of its facets (*p* < 0.01, cluster threshold estimator corrected). From top to bottom: **(A)** FFMQ_Total_; **(B)** *Observing*; **(C)** *Describing*; **(D)** *Acting*; **(E)** *Non-Judging*; **(F)** *Non-Reactivity*.

The significant clusters found in the six ANCOVAs assessing the functional connectivity of the component featuring the DMN are listed in Table 2 (see Supplementary Materials for cluster locations described as MNI coordinates). Overall trait mindfulness scores (FFMQ_Total_) correlated with four clusters in the DMN (Figure 1A). The first cluster positively correlated with FFMQ_Total_ (*r* = 0.67, *p* < 0.01) and was located primarily in the anterior right hemisphere. The regions comprising this cluster included the anterior cingulate, mid-cingulate gyrus, caudate, and medial frontal gyrus. The second cluster negatively correlated with FFMQ_Total_ (*r* = −0.74, *p* < 0.01) and was focused on the left middle and inferior temporal gyri and the left middle and superior occipital gyri. The third cluster negatively correlated with FFMQ_Total_ (*r* = −0.73, *p* < 0.01) and was located in similar regions to the second cluster, except in the right hemisphere. The fourth cluster negatively correlated with FFMQ_Total_ (*r* = −0.77, *p* < 0.01) and was located in the right middle temporal and superior temporal gyri.

**Table 2:** Talairach coordinates of correlations between FFMQ scores and functional connectivity with the default mode network (DMN).

			Talairach coordinates						
Region	Hemisphere	Gyrus	BA	*X*	*Y*	*Z*	Cluster size	*r*	*p*
**Trait mindfulness (FFMQ_Total_)**									
Limbic	Right	Anterior cingulate	32	8	37	24	10,757	0.67	0.000082
Temporal	Left	Middle temporal	39	−46	−74	12	6,350	−0.74	0.000008
	Right	Middle temporal	37	56	−65	3	3,312	−0.73	0.000012
	Right	Superior temporal	21	53	−5	−12	2,525	−0.77	0.000012
**Observing**									
Frontal	Right	Precentral gyrus	44	59	7	12	1,829	−0.67	0.000086
**Describing**									
Parietal	Right	Superior parietal	7	26	−63	66	9,435	−0.69	0.000056
**Acting**									
Sub-lobar	Right	Caudate	CH	20	22	0	2,392	0.62	0.000388
Occipital	Right	Cuneus	18	5	−98	3	1,647	0.63	0.000327
Frontal	Right	Superior frontal	6	5	37	61	5,209	−0.67	0.000107
Temporal	Left	Middle temporal	21	−55	7	−24	2,507	−0.68	0.000065
Posterior lobe	Left	Inferior semi-lunar	*	−19	−74	−39	2,269	−0.64	0.000272
**Non-judging**									
Limbic	Right	Cingulate gyrus	24	2	−8	36	2,247	0.59	0.001074
**Non-reactivity**									
Posterior lobe	Right	Cerebellar tonsil	*	41	−44	−39	2,705	0.64	0.000238
Temporal	Right	Superior temporal	22	56	−2	−3	6,429	−0.74	0.000006
	Right	Superior temporal	22	50	−47	15	2,931	−0.63	0.000367
	Left	Middle temporal	39	−40	−68	15	3,907	−0.69	0.000053

Abbreviations: BA, Brodmann Area; CH, caudate head; *region not affiliated with a BA.

*Observing* scores negatively correlated with one cluster in the component featuring the DMN (*r* = −0.67, *p* < 0.01; Figure 1B). Regions comprising this cluster included the right inferior frontal and precentral gyri.

*Describing* scores were also negatively correlated with one cluster in the DMN component (*r* = −0.69, *p* < 0.01; Figure 1C). This cluster was located in the left and right precuneus and superior parietal lobule.

*Acting* correlated with five clustersData from one participan in the DMN component (Figure 1D). The first cluster positively correlated with *Acting* (*r* = 0.62, *p* < 0.01) and was focused in the right lentiform nucleus, caudate, and claustrum. The second cluster positively correlated with *Acting* (*r* = 0.63, *p* < 0.01) and was located in the right and left cuneus and lingual gyrus. The third cluster negatively correlated with *Acting* (*r* = −0.67, *p* < 0.01) and appeared in the right superior and middle frontal gyri and sub-gyral frontal lobe. The fourth cluster also negatively correlated with *Acting* (*r* = −0.68, *p* < 0.01) and appeared primarily in the left middle, superior, and inferior temporal gyri, with some representation in the left fusiform gyrus and sub-gyral temporal lobe. The fifth cluster negatively correlated with *Acting* (*r* = −0.64, *p* < 0.01) and was located in the left inferior semi-lunar lobule and pyramis.

*Non-Judging* scores were positively correlated with one cluster in the DMN component (*r* = 0.59, *p* < 0.01; Figure 1E). This cluster was focused entirely on the right and left cingulate gyri (Brodmann Area 24).

*Non-Reactivity* scores correlated with four clusters in the DMN component (Figure 1F). The first cluster positively correlated with *Non-Reactivity* (*r* = 0.64, *p* < 0.01) and appeared in the right cerebellar tonsil and the right fusiform and inferior temporal gyri. The remaining three clusters were negatively correlated with *Non-Reactivity*. One cluster was focused within the right superior temporal gyrus (STG), extending slightly into the middle and transverse temporal gyri and the precentral gyrus (*r* = −0.74, *p* < 0.01). The second negatively correlated cluster included the right superior and middle temporal gyri and the insula (*r* = −0.63, *p* < 0.01). The remaining negative cluster was located primarily in the left middle temporal gyrus, with smaller areas in the superior temporal and middle occipital gyri (*r* = −0.69, *p* < 0.01).

### Salience Network Functional Connectivity

**Figure 2:**
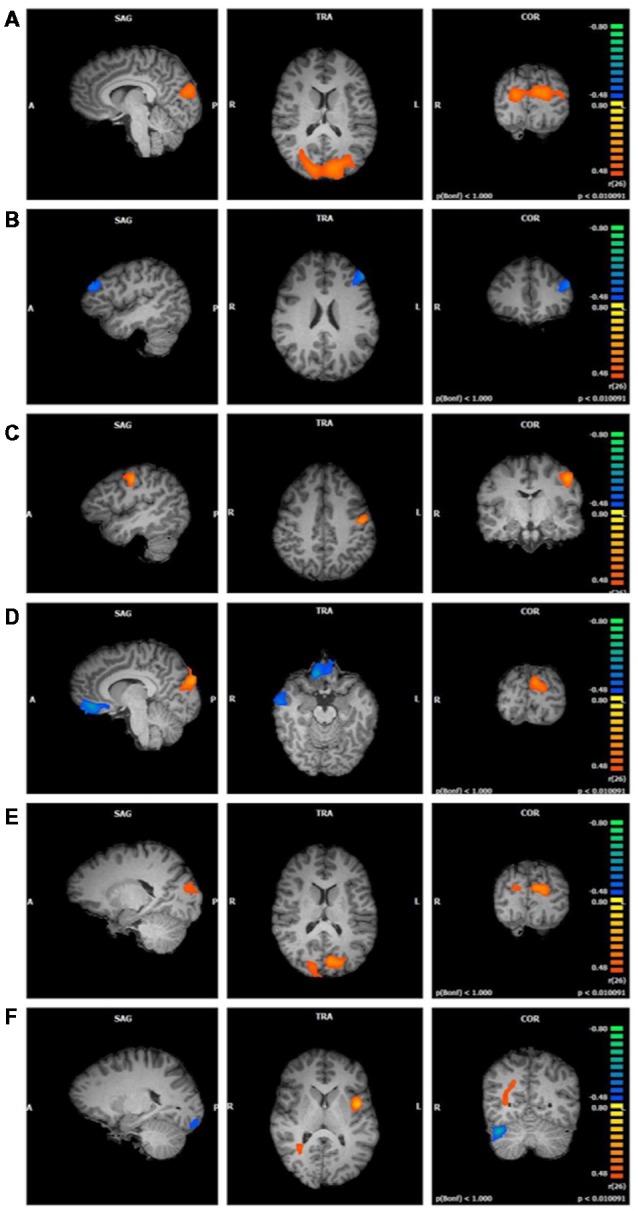
Functional connectivity of salience network (SN) clusters correlated with trait mindfulness and five of its facets (*p* < 0.01, cluster threshold estimator corrected). From top to bottom: **(A)** FFMQ_Total_; **(B)** *Observing*; **(C)** *Describing*; **(D)** *Acting*; **(E)** *Non-Judging*; **(F)** *Non-Reactivity*.

The significant clusters found in the six ANCOVAs assessing the functional connectivity of the component featuring the SN are listed in Table 3. FFMQ_Total_ positively correlated with one cluster in the SN (*r* = 0.78, *p* < 0.01). This cluster was focused in the right and left cuneus, with smaller regions in the middle occipital gyrus and precuneus (Figure 2A).

**Table 3:** Talairach coordinates of correlations between FFMQ scores and functional connectivity with the salience network (SN).

			Talairach coordinates						
Region	Hemisphere	Gyrus	BA	*X*	*Y*	*Z*	Cluster size	*r*	*p*
**Trait Mindfulness (FFMQ_Total)**									
Occipital	Right	Cuneus	18	17	−83	12	21,643	0.78	0.000001
**Observing**									
Frontal	Left	Middle frontal	46	−49	41	27	2,966	−0.64	0.000282
**Describing**									
Frontal	Left	Precentral gyrus	4	−49	−11	45	2,691	0.66	0.000129
**Acting**									
Occipital	Left	Cuneus	19	−7	−92	24	6,940	0.68	0.000077
Frontal	Right	Rectal gyrus	11	11	31	−19	7,983	−0.68	0.000066
Temporal	Right	Fusiform gyrus	20	59	−5	−24	3,947	−0.69	0.000054
**Non-judging**									
Occipital	Left	Cuneus	18	−7	−77	18	3,249	0.65	0.0002
	Right	Cuneus	18	17	−83	18	2,133	0.61	0.000517
**Non-reactivity**									
Frontal	Left	Precentral gyrus	44	−43	1	9	3,023	0.71	0.000025
No gray matter found			*	23	−50	27	2,390	0.55	0.00244
Posterior lobe	Right	Tuber	*	38	−56	−30	4,338	−0.69	0.000048
Occipital	Left	Lingual gyrus	17	−13	−95	−18	2,502	−0.65	0.0002

Abbreviations: BA, Brodmann Area; *region not affiliated with a BA.

*Observing* scores were negatively correlated with one cluster in the SN component (*r* = −0.64, *p* < 0.01; Figure 2B). Regions comprising this cluster included the left middle and superior frontal gyri.

*Describing* positively correlated with one cluster in the SN component (*r* = 0.66, *p* < 0.01; Figure 2C). This cluster was located in the left pre- and post-central gyri.

*Acting* values were correlated with three clusters in the SN component (Figure 2D). The first cluster positively correlated with *Acting* (*r* = 0.68, *p* < 0.01) in the right and left cuneus and middle occipital gyrus. The second cluster negatively correlated with *Acting* (*r* = −0.68, *p* < 0.01) and included bilateral regions of the medial and inferior frontal gyri, anterior cingulate gyrus, and rectal gyrus. The third cluster negatively correlated with *Acting* (*r* = −0.69, *p* < 0.01) and was located in the right inferior, middle, and superior temporal gyri and fusiform gyrus.

*Non-Judging* scores were positively correlated with two clusters in the SN component, one in the left hemisphere and the other in right hemisphere (first cluster *r* = 0.65, *p* < 0.01; second cluster *r* = 0.61, *p* < 0.01; Figure 2E). These clusters were similar, with the cuneus comprising the majority of each cluster and a smaller portion in the middle occipital gyrus.

*Non-Reactivity* values were correlated with four clusters in the SN component (Figure 2F). The first cluster positively correlated with *Non-Reactivity* (*r* = 0.71, *p* < 0.01). This cluster was located in the left insula, with smaller portions extending into the precentral gyrus, STG, and inferior frontal gyrus. The second cluster was also positively correlated with *Non-Reactivity* (*r* = 0.55, *p* < 0.01); however, it was not located in gray matter. The third and fourth clusters were negatively correlated with *Non-Reactivity* scores. One of these clusters was located in the right cerebellum (*r* = −0.69, *p* < 0.01). The cerebellar tonsil and tuber comprised the majority of this cluster, with smaller areas in the culmen, declive, and anterior lobe. The other negatively correlated cluster consisted primarily of white matter; the gray matter regions included the left uvula and declive in the cerebellum as well as the left fusiform lingual gyri (*r* = −0.65, *p* < 0.01).

### Central Executive Network Functional Connectivity

**Figure 3:**
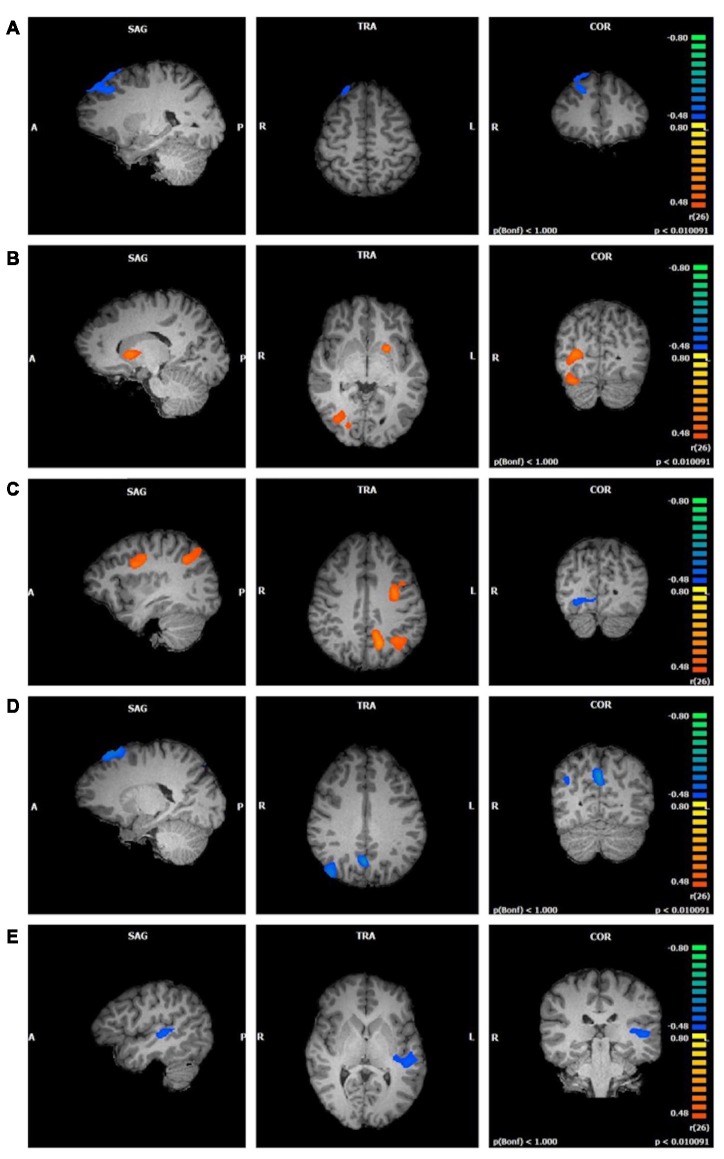
Functional connectivity of central executive network (CEN) clusters correlated with trait mindfulness and four of its facets (*p* < 0.01, cluster threshold estimator corrected). From top to bottom: **(A)** FFMQ_Total_; **(B)** *Observing*; **(C)** *Describing*; **(D)** *Acting*; **(E)** *Non-Reactivity*.

The significant clusters found in the six ANCOVAs assessing the functional connectivity of the component featuring the CEN are listed in Table 4. FFMQ_Total_ negatively correlated with one cluster in the CEN component (*r* = −0.60, *p* < 0.01; Figure 3A). Only a small portion of this cluster was located in gray matter and included the right middle and superior frontal gyri.

**Table 4:** Talairach coordinates of correlations between FFMQ scores and functional connectivity with the central executive network (CEN).

			Talairach coordinates						
Region	Hemisphere	Gyrus	BA	*X*	*Y*	*Z*	Cluster size	*r*	*p*
**Trait Mindfulness (FFMQ_Total)**									
Frontal	Right	Superior frontal	6	26	34	61	3,490	−0.60	0.000805
**Observing**									
Occipital	Right	Lingual gyrus	19	29	−68	3	2,961	0.76	0.000002
Sub-lobar	Left	Lentiform nucleus	P	−22	7	0	2,872	0.64	0.000261
Posterior lobe	Right	Uvula	*	29	−77	−24	2,256	0.63	0.00035
**Describing**									
Frontal	Left	Precentral gyrus	6	−31	−2	33	31,308	0.65	0.000202
Parietal	Left	Precuneus	7	13	−62	36	17,808	0.67	0.000109
Occipital	Right	Fusiform gyrus	19	26	−74	−12	18,108	−0.58	0.00114
**Acting**									
No gray matter found			*	17	28	63	3,548	−0.71	0.000026
Occipital	Right	Precuneus	31	2	−62	27	2,315	−0.65	0.000159
Parietal	Right	Precuneus	19	38	−80	37	2,262	−0.59	0.00107
**Non-reactivity**									
Temporal	Left	Superior temporal	41	−46	−29	3	2,229	−0.60	0.000718

Abbreviations: BA, Brodmann Area; P, putamen; *region not affiliated with a BA.

*Observing* scores were positively correlated with three clusters in the CEN component (first cluster *r* = 0.76; second cluster *r* = 0.64; third cluster *r* = 0.63; *p* < 0.01; Figure 3B). The first cluster, although the largest, contained the smallest representation of regions in gray matter. This cluster was located in the right lingual gyrus with a small portion extending into the right posterior cingulate. The second cluster was located primarily in the left lentiform nucleus, with a smaller portion in the left caudate. The third cluster spanned regions in the right cerebellum including the uvula, tuber, declive, and pyramis.

*Describing* correlated with three large clusters in the CEN component (Figure 3C). The first cluster positively correlated with *Describing* (*r* = 0.65, *p* < 0.01) and included bilateral regions of the superior, middle, and medial frontal gyri, precentral gyrus, and posterior cingulate gyrus. The second cluster positively correlated with *Describing* (*r* = 0.67, *p* < 0.01) and was focused on the left cerebrum. The left precuneus, left inferior and superior parietal lobules, and the left posterior cingulate and angular gyri represented this cluster. The third cluster negatively correlated with *Describing* (*r* = −0.58, *p* < 0.01) and was located in the right declive and culmen in the cerebellum, as well as the right lingual, fusiform, and posterior cingulate gyri.

*Acting* was negatively correlated with three clusters in the CEN component (first cluster *r* = −0.71; second cluster *r* = −0.65; third cluster *r* = −0.59; *p* < 0.01; Figure 3D). The first cluster appeared primarily in white matter, with the right superior and middle frontal gyri comprising the gray matter areas. The second cluster was located in the left and right precuneus, cuneus, and posterior cingulate gyrus, regions typically associated with the DMN. The third cluster was focused in the right precuneus, with the right angular, superior occipital, and middle temporal gyri representing smaller portions.

There was no relationship between *Non-Judging* and the CEN component. *Non-Reactivity*, on the other hand, negatively correlated with one cluster in the CEN component (*r* = −0.60, *p* < 0.01; Figure 3E). This cluster was located in the left superior, middle, and transverse temporal gyri, claustrum, and insula.

### Attention Network Functional Connectivity

The significant clusters found in the six ANCOVAs assessing the functional connectivity of the component featuring the ATN are listed in Table 5.

**Table 5:** Talairach coordinates of correlations between FFMQ scores and functional connectivity with the attentional network (ATN).

			Talairach coordinates						
Region	Hemisphere	Gyrus	BA	*X*	*Y*	*Z*	Cluster size	*r*	*p*
**Observing**									
Frontal	Right	Middle frontal	9	35	13	27	5,150	0.70	0.000035
Sub-lobar	Left	Insula	13	−40	1	21	3,037	0.75	0.000004
Parietal	Left	Supramarginal	40	−65	−50	36	2,141	−0.61	0.000592
**Describing**									
Temporal	Right	Superior temporal	22	47	−20	−6	1,731	−0.67	0.000094
	Left	Superior temporal	22	−49	−44	12	1,638	−0.68	0.000066
**Acting**									
Occipital	Right	Lingual gyrus	19	17	−56	0	3,256	−0.69	0.000051
**Non-judging**									
Frontal	Right	Superior frontal	9	11	58	27	1,686	0.63	0.00031

*Abbreviations: BA, Brodmann Area*.

**Figure 4:**
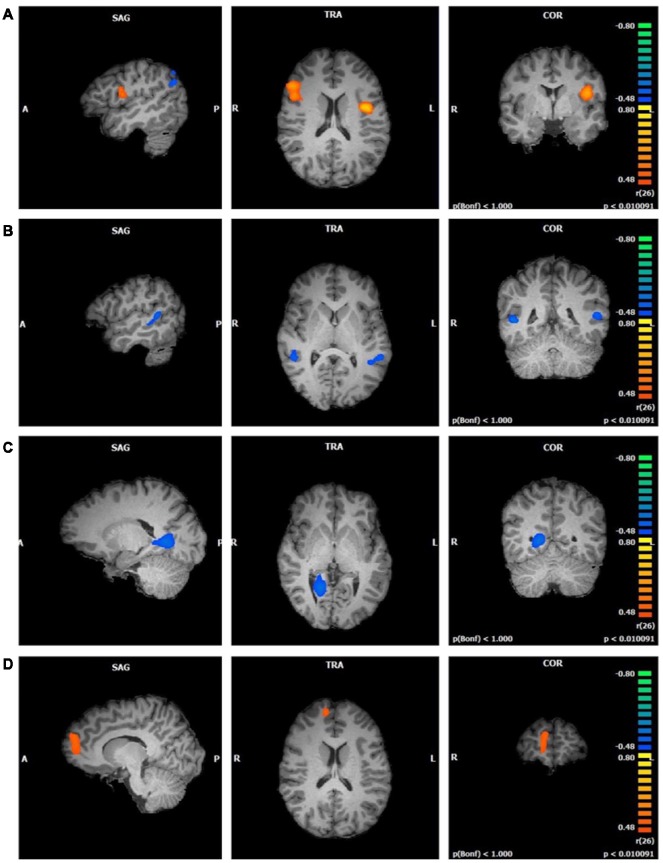
Functional connectivity of attentional network (ATN) clusters correlated with four mindfulness facets (*p* < 0.01, cluster threshold estimator corrected). From top to bottom: **(A)** *Observing*; **(B)** *Describing*; **(C)** *Acting*; **(D)** *Non-Judging*.

There was no relationship between FFMQ_Total_ and the ATN component. *Observing* scores were correlated with three clusters in the ATN component. The first cluster positively correlated with *Observing* (*r* = 0.70, *p* < 0.01; Figure 4A) and was located in the right middle, inferior, and superior frontal gyri. The second cluster positively correlated with *Observing* (*r* = 0.75, *p* < 0.01) and was located in the left insula, precentral gyrus, and inferior frontal gyrus. The third cluster negatively correlated with *Observing* (*r* = −0.61, *p* < 0.01) and included the left supramarginal and angular gyri, inferior parietal lobule, and middle and superior temporal gyri.

*Describing* values were negatively correlated with two clusters in the ATN component (first cluster *r* = −0.67; second cluster *r* = −0.68; *p* < 0.01; Figure 4B). These clusters were similar in size and were both located in the superior and middle temporal gyri. The first cluster comprised these regions in the right hemisphere and the second cluster in the left hemisphere.

*Acting* was negatively correlated with one cluster in the ATN component (*r* = −0.69, *p* < 0.01; Figure 4C). The right lingual gyrus comprised most of this cluster, with smaller areas in the right parahippocampal gyrus, posterior cingulate, and culmen.

*Non-Judging* scores were positively correlated with one cluster in the ATN component (*r* = 0.63, *p* < 0.01; Figure 4D). This cluster was located in the right superior and medial frontal gyri. There was no relationship between *Non-Reactivity* values and the ATN.

## Discussion

The results of this study indicate that trait mindfulness influences functional connectivity in four resting state networks associated with cognition and attention. The following discussion provides several interpretations for the observed patterns of functional connectivity. However, it is important to advise caution in linking the results of functional connectivity studies to specific cognitive processes or behavioral patterns. Given that attentional or sensory processes were not directly tested in this study, stating that the significant voxels detected were associated with a specific cognitive or attentional process would be a “reverse inference” error (Poldrack, 2006). We will, therefore, focus on specific trends in the data, focusing on how the current results relate to previous studies of mindfulness and on how our data could be used to generate hypotheses in future task-based studies.

### Trait Mindfulness

FFMQ_Total_ scores were related to the functional connectivity of two MPFC regions. The component featuring the DMN demonstrated increased functional connectivity in the right ACC for individuals scoring higher in trait mindfulness. This finding is consistent with previous structural neuroimaging studies (Tang et al., 2010, 2012). Tang et al. (2010, 2012) reported that a specific form of mindfulness training (Integrative Body-Mind Training, IBMT) increased the efficiency of white matter tract functioning—as shown by an increase in fractional anisotropy (FA) and decreases in both axial and radial diffusivity—between the ACC and its connecting structures. This increase in FA corresponds to a strengthening of ACC connections, with a possible association for enhanced self-regulation (Tang et al., 2010). These results complement a task-based study from Kilpatrick et al. (2011). Following an 8-week Mindfulness-Based Stress Reduction (MBSR) program, functional connectivity changed during a practice of state mindfulness in female meditation-naïve participants. They reported increased functional connectivity between the DMPFC and dorsal ACC, and suggested that this change was linked with a greater awareness of attentional and sensory experiences rather than engaging mainly in self-referential reflection (Kilpatrick et al., 2011).

FFMQ_Total_ scores were also linked with altered functional connectivity in the DMPFC in the CEN component. The CEN component demonstrated reduced functional connectivity in the right superior frontal gyrus (SFG), located in the DMPFC. The DMPFC is a region of the DMN that is associated with weaker functional connectivity in experienced meditators (Taylor et al., 2013). Taylor et al. (2013) suggest that novice meditators use cortical structures to down-regulate regions related to emotional processing, such as the amygdala, whereas experienced meditators exhibit emotional stability and acceptance that do not require the control of affective systems by cortical structures. The current results suggest that individuals who score high on measures of trait mindfulness have patterns of functional connectivity that resemble (to some degree) experienced meditators. This conclusion would be consistent with other studies suggesting that trait mindfulness uses less input from medial cortical structures (Farb et al., 2007, 2012).

### Facets of Trait Mindfulness

For the *Observing* facet, the ATN component demonstrated increased functional connectivity in the insula, a SN structure that is involved with interoceptive awareness (Critchley et al., 2004; Farb et al., 2013). Bilevicius et al. (2018) observed a similar result in their study of the MAAS, reporting a positive association between the attentional aspect of trait mindfulness and the left insula in the SN. Murakami et al. (2012) analyzed how gray matter volume correlated with a Japanese version of the FFMQ. Interestingly, they found that the right anterior insula positively associated with the *Describing* facet. While these results are not identical, they do indicate that the functional connectivity of the insula is related to multiple facets of trait mindfulness, and suggest that the insula should be a region of interest in future seed-based studies of functional connectivity and trait mindfulness.

The DMN component showed increased functional connectivity with the right mid-cingulate gyrus when its connectivity was co-varied with *Non-Judging* scores. This region is associated with the anticipation of pain (Brown and Jones, 2008). The practice of mindfulness brings non-judgmental attention toward painful stimuli in an effort to decrease pain perception (Gard et al., 2011). It has also been shown that mindfulness practitioners rate pain as less unpleasant relative to controls (Gard et al., 2011). Brown and Jones (2008) suggest that mid-cingulate areas direct attentional resources toward a painful stimulus to reduce focus on the emotional aspect of pain and to increase focus on the sensory experience (Brown and Jones, 2008). In the present study, the DMN component correlating with the mid-cingulate gyrus for *Non-Judging* may be associated with a more sensory and less cognitive, self-referential process. However, behavioral studies would be necessary to test whether the functional connectivity between the mid-cingulate gyrus and the DMN is specifically related to altered nociceptive responses.

When co-varied with *Non-Judging* scores, the ATN component showed increased functional connectivity with the right anteromedial SFG, a region located in the rostral MPFC that is a node of the DMN and which connects to nodes in the SN (Ongür and Price, 2000). Other studies have reported increased functional connectivity with the MPFC in the DMN in meditators relative to controls (Jang et al., 2011; Hasenkamp and Barsalou, 2012). Hasenkamp and Barsalou (2012) suggested that experienced meditators show greater connectivity to this DMN node, thus reflecting a greater level of awareness of their internal states. In a study comparing experienced meditators and novice participants, Froeliger et al. (2012) demonstrated increased resting-state functional connectivity between the DAN (a network of the ATN) and the DMN as well as between the DAN and the right anterior PFC. These researchers suggested that these patterns of connectivity represented a greater allocation of resources towards attention and awareness (Froeliger et al., 2012). The current study suggests that this relationship may be specifically related to the *Non-Judging* facet of mindfulness.

### Combined Trends

In analyzing the ICA results across resting state networks and mindfulness facets, four overarching patterns emerged. First, the components featuring two of the cognitive networks correlated with an additional sensory region (i.e., an area that is not typically associated with the network). The precentral gyrus, a region related to sensorimotor processing, correlated with the SN (*Describing*, *Non-Reactivity*) and CEN (*Describing*) components. Farb et al. (2012) suggest that mindfulness training enhances emotional regulation by transitioning from a cognitive focus to a present-moment awareness using the thalamus, insula, and sensorimotor regions. The present study provides support for this notion, demonstrating that individuals higher in trait mindfulness engage the insula and sensorimotor regions. Further behavioral studies would be required to examine the possibility that the correlation of sensorimotor nodes in resting state networks may be related to enhanced emotional regulation and a present-moment focus. If so, this would be consistent with two of the goals of mindfulness: to achieve greater emotional regulation and self-awareness.

Second, the components featuring the three cognitive networks correlated with two nodes related to visual processing. The cuneus, a region implicated in visual processing (Vanni et al., 2001), correlated with the DMN (*Acting*) and SN (FFMQ_Total_, *Acting*, *Non-Judging*) components. The lingual gyrus, a region involved in color vision and dreaming (Corbetta et al., 1990; Domhoff and Fox, 2015), correlated with the CEN (*Observing*) component. This pattern of data may suggest that individuals with higher trait mindfulness more efficiently allocate neural resources during visual perception. This idea is indirectly supported by other studies identifying increased visual sensitivity (Brown et al., 1984) and vigilance (MacLean et al., 2010) following a 3-month intensive meditation program. Enhanced vigilance following mindfulness training appeared to be adaptive for attentional tasks in that fewer resources were required to visually discriminate a stimulus, thus allowing for enhanced attention and concentration (MacLean et al., 2010). Whether the perceptual changes associated with mindfulness training are related to changes in functional connectivity similar to those observed in the current study is an intriguing avenue for future investigations.

Third, two cognitive network components, the DMN and CEN, positively correlated with the cerebellum when co-varied with the *Non-Reactivity* and *Observing* facets, respectively. The cerebellum, while mainly known for its functions in motor control, has also been implicated in higher cognitive functions including the regulation of affect and cognition (Ramnani, 2006; Stoodley and Schmahmann, 2010). The DMN and CEN components associated with Crus I and lobule VI, respectively, regions in the right posterior lobe that are associated with language and executive functioning (Stoodley and Schmahmann, 2009). Hölzel et al. (2011) found that following the completion of an MBSR program, participants demonstrated an increase in gray matter concentration in the vermis and posterior cerebellar lobe, regions associated with the regulation of cognitive processes such as perception and thinking. One of the benefits of mindfulness is the regulation of cognition for healthy psychological functioning; thus, it is possible that the DMN and CEN correlating with these cerebellar regions indicates their involvement in these processes for those high in trait mindfulness.

Finally, the STG was found to be consistently negatively correlated across mindfulness facets. The anterior and posterior STG demonstrated reduced functional connectivity within cognitive and attentional (DMN, CEN, and ATN) network components. The STG is a primary node of the auditory network, with the posterior region associated with the perception of speech sounds (Chang et al., 2010) and the anterior region involved in the semantic processing of auditory and visual information (Visser and Lambon Ralph, 2011). Visser and Lambon Ralph (2011) provide evidence that the anterior temporal lobe (which includes the anterior STG) represents a hub for visual and sensory information that is activated during semantic processing of these modalities. Given that these regions are associated with active semantic processing, it seems likely that the STG would show widespread reduced connectivity during a resting state. Moreover, the reduced correlation between trait mindfulness and the STG is consistent with Bilevicius et al.’s (2018) study examining the relationship between functional connectivity and scores on the MAAS. These authors reported that the DMN and bilateral CEN demonstrated reduced connectivity between trait mindfulness and the STG (Bilevicius et al., 2018).

Collectively, these data indicate an overall greater integration of attentional, sensory, and interoceptive neural regions for individuals higher in trait mindfulness and its facets. This may be related to individuals higher in trait mindfulness demonstrating a more present-moment focus and enhanced emotional regulation, with less emphasis on self-reference.

### Relationship to Lesion Studies

The correlative nature of resting-state fMRI makes it impossible to state that changes in functional connectivity *cause* a specific trait such as mindfulness (Poldrack, 2006). It is therefore interesting to examine whether other studies have identified impairments in mindfulness-like functions (e.g., emotional regulation) in patients who have experienced lesions in the brain areas highlighted in the current study. Such data would help reinforce the fact that many of the brain areas discussed in the current study do perform functions related to mindfulness; however, it is important to reiterate that the current data cannot be linked to specific functions.

The data from the current study indicate that individual differences in trait mindfulness are related to differences in functional connectivity in regions of the cerebellum, MPFC, posterior temporal lobe, anterior cingulate/insula, and precuneus. Previous patient-based research has found that cerebellar damage—particularly to posterior and medial regions—leads to impairments in executive control and emotional regulation (Schmahmann and Sherman, 1998). These functions are key facets of mindfulness. Notably, functional connectivity studies have found that the cerebellum has distinct connections with the medial and DLPFC (Krienen and Buckner, 2009). Damage to these prefrontal regions has been implicated in social cognition, self-relevant processing, and emotional perception (Hornak et al., 2003; for a review, see Lieberman et al., 2019). Similar impairments have been noted in patients with damage to the posterior regions of the temporal lobes (Campanella et al., 2014). Additional studies have noted that damage to the temporoparietal regions also leads to deficits in bodily awareness (Martinaud et al., 2017) and language comprehension (e.g., Benghanem et al., 2019), which would include the ability to describe one’s experiences. Again, these functions are related to present-moment awareness of one’s thoughts and bodily experiences. Lesions to the insula have been linked with alterations in sensitivity to somatosensory information (Karnath et al., 2005; for a review, see Craig, 2009). In contrast, the functional consequences of lesions to the precuneus are more difficult to identify, with damage to different subregions leading to different forms of impairments (Harroud et al., 2017). It is worth noting, however, that meditation training has been linked with increases in the volume of this region (Kurth et al., 2014).

Taken together, these lesion data suggest that the brain areas identified in the current study are related to behaviors that are associated with mindfulness (e.g., emotional regulation, interoception, attentional control). However, additional task-based fMRI investigating these behaviors in individuals who are high or low on trait mindfulness is necessary to help corroborate these speculations.

### Limitations and Future Directions

Although the current research provides novel information about how the functional connectivity of four resting-state networks could be related to different facets of trait mindfulness, there are many limitations that could be addressed in future studies. One limitation to this study is the use of the ICA approach for statistical analyses. This approach, given the exploratory nature, may not be used to directly test hypotheses. Future research could employ other approaches to resting state fMRI analysis. For example, graph theory (although similar to ICA in that it is also data-driven) measures the local and global organization of the neural networks (Wang et al., 2010). Graph theory analysis would provide information regarding the topology of the brain networks and may provide further insight into the integration of nodes in different networks as a function of trait mindfulness. Alternately, hypothesis-driven analyses, such as seed-based or region-of-interest analyses, could be used to develop functional connectivity maps (e.g., of the insula or ACC) that examine how the functional connectivity of specific regions relate to trait mindfulness. A further limitation with the ICA approach is that the DAN and VAN networks were contained within the same component. Although both networks have complementary attention-related functions, it was not possible to discern whether their relationship with FFMQ facets differed with this method. Additionally, given that there was no cognitive task used in this study, it is problematic to relate the current data to specific cognitive, attentional, or emotional behaviors. Further experimentation using task-based fMRI would allow researchers to more definitively state whether a specific brain area was involved in a precise cognitive or interoceptive function related to mindfulness.

Additional limitations relate to variables that could have influenced the relationship between trait mindfulness and fluctuations in neural activity. First, additional personality questionnaires could have been administered to participants in order to determine whether these traits co-varied with the different facets of trait mindfulness. Second, although the exclusion criteria for this study precluded participants with psychiatric illness, it is possible that some people may have had sub-clinical symptoms. Future studies may include a standardized measure to objectively screen for underlying symptoms. Finally, information on participants’ thought processes during the MRI scan was not collected. Future studies may provide this information to identify if patterns of functional connectivity are consistent with a particular mental state.

It is also important to note that while participants were meditation naïve, it is possible that they had other meditation-like experiences, such as through yoga or prayer. This issue is important because statements on the FFMQ related to the *Observing* facet may be interpreted differently between meditators and non-meditators (Williams et al., 2014). Williams et al. (2014) found that a four-factor hierarchical model of the FFMQ (without the *Observing* category) was a better fit than a five-factor model for both clinical and non-clinical samples. They suggested that the *Describing*, *Acting*, *Non-judging*, and *Non-reactivity* facets are important to well-being for non-meditators, and with increasing meditation experience the *Observing* facet becomes more important (Williams et al., 2014). If the *Observing* facet is less important at earlier stages of meditation training, the statements may be interpreted differently. Thus, some caution must be used when interpreting the results of this facet of mindfulness. Additional research is necessary in order to compare the cognitive and neural underpinnings of yoga, prayer, and different forms of meditation, and to determine if they affect functional connectivity in a manner similar to trait (or state) mindfulness.

## Conclusion

The purpose of the current study was to provide novel information about the neural substrates of trait mindfulness, highlighting how individual differences in the functional connectivity of cognitive and attentional resting state networks are related to different facets of this beneficial personality trait. The analyses noted functional connectivity in the ACC and insula, regions that have previously been linked to attentional control and interoception. The analyses also showed interesting mindfulness-dependent variability in the connectivity of the mid-cingulate gyrus, cerebellum, and sensorimotor regions. Together, these results suggest that trait mindfulness is related to the functional connectivity of neural regions involved with cognition, emotion, *and* sensation. Trait mindfulness involves all of these processes on some level, from attending to present-moment internal and external sensations, to reducing rumination and self-referential thought processes, to regulating emotions. Overall, the correlation with functional areas associated with these processes suggests that focusing on the present moment in a non-judgmental fashion may allow for the integration of multisensory modalities to facilitate greater self-awareness.

## Ethics Statement

The University of Winnipeg Human Research Ethics Board and the Bannatyne Human Research Ethics Board provided ethical approval for this study.

## Author Contributions

SS and JK designed the study. SS was present for the MRI scans and provided the FFMQ to participants. TP analyzed the data and wrote the manuscript, with support from SS and JK.

## Conflict of Interest Statement

The authors declare that the research was conducted in the absence of any commercial or financial relationships that could be construed as a potential conflict of interest.
