# Supplementary material for: Trait Mindfulness and Functional Connectivity in Cognitive and Attentional Resting State Networks
Source: Front Hum Neurosci. 2019 Apr 12;13:112. doi: 10.3389/fnhum.2019.00112 (PMC6473082; doi:10.3389/fnhum.2019.00112)
Supplement: Supplementary file 1 [file Table_1.pdf]

Supplementary Materials

The following tables contain the Montreal Neurological Institute (MNI) coordinates for the data reported in the manuscript:

Parkinson TD, Kornelsen J, and Smith SD (2019). Trait Mindfulness and Functional Connectivity in Cognitive and Attentional Resting State Networks. *Front. Hum. Neurosci.* 13:112. doi: 10.3389/fnhum.2019.00112

Table 1 from this manuscript consists of information about the participants; it is not reproduced in the Supplementary Materials. Tables 2-5 contain data related to functional connectivity analyses; these Tables are reproduced here with MNI coordinates replacing the Talairach coordinates used in the published manuscript.

We have kept the names of the Tables in the Supplementary Materials identical to those tables in the main manuscript (e.g., Table 2 in the Supplementary Materials contains MNI coordinates for the data in Table 2 of the main manuscript). Therefore, the current file will contain only Tables 2-5 (as Table 1 only contained participant information).

Table 2. Coordinates of correlations between FFMQ scores and functional connectivity with the DMN.

Region	Hemisphere	Gyrus	MNI Coordinates[1]						
			BA	X	Y	Z	Cluster size	*r*	*p*
**Trait Mindfulness (FFMQ$_{Total}$)**									
Limbic	Right	Anterior cingulate	32	9	38	26	10757	0.67	0.000082
Temporal	Left	Middle temporal	39	-46	-76	9	6350	-0.74	0.000008
	Right	Middle temporal	37	56	-65	-2	3312	-0.73	0.000012
	Right	Superior temporal	21	56	-2	-19	2525	-0.77	0.000012
**Observing**									
Frontal	Right	Precentral gyrus	44	62	9	9	1829	-0.67	0.000086
**Describing**									
Parietal	Right	Superior parietal	7	25	-69	75	9435	-0.69	0.000056
**Acting**									
Sub-lobar	Right	Caudate	CH	21	25	-3	2392	0.62	0.000388
Occipital	Right	Cuneus	18	5	-102	-1	1647	0.63	0.000327
Frontal	Right	Superior frontal	6	5	33	68	5209	-0.67	0.000107
Temporal	Left	Middle temporal	21	-57	13	-31	2507	-0.68	0.000065
Posterior lobe	Left	Inferior semi-lunar	*	-20	-72	-50	2269	-0.64	0.000272
**Non-judging**									
Limbic	Right	Cingulate gyrus	24	2	-10	38	2247	0.59	0.001074
**Non-reactivity**									
Posterior lobe	Right	Cerebellar tonsil	*	42	-41	-50	2705	0.64	0.000238
Temporal	Right	Superior temporal	22	60	1	-9	6429	-0.74	0.000006
	Right	Superior temporal	22	51	-48	13	2931	-0.63	0.000367
	Left	Middle temporal	39	-40	-70	12	3907	-0.69	0.000053

Abbreviations: BA = Brodmann Area; CH = caudate head; * = region not affiliated with a BA; [1] the MNI coordinates reported here are a direct conversion of the Talairach coordinates presented in the paper using the Yale BioImage Suite (1.0.0, 2018/12/12) online tool found at [https://bioimagesuiteweb.github.io/webapp/mni2tal.html].

Table 3. Coordinates of correlations between FFMQ scores and functional connectivity with the SN.

Region	Hemisphere	Gyrus	MNI Coordinates[1] BA	X	Y	Z	Cluster size	*r*	*p*
**Trait Mindfulness (FFMQ$_{Total}$)**									
Occipital	Right	Cuneus	18	17	-86	9	21643	0.78	0.000001
**Observing**									
Frontal	Left	Middle frontal	46	-50	41	29	2966	-0.64	0.000282
**Describing**									
Frontal	Left	Precentral gyrus	4	-50	9	48	2691	0.66	0.000129
**Acting**									
Occipital	Left	Cuneus	19	-7	-97	24	6940	0.68	0.000077
Frontal	Right	Rectal gyrus	11	12	45	-20	7983	-0.68	0.000066
Temporal	Right	Fusiform gyrus	20	62	-1	-32	3947	-0.69	0.000054
**Non-judging**									
Occipital	Left	Cuneus	18	-7	-81	17	3249	0.65	0.0002
	Right	Cuneus	18	17	-87	17	2133	0.61	0.000517
**Non-reactivity**									
Frontal	Left	Precentral gyrus	44	-45	4	7	3023	0.71	0.000025
No grey matter found			*	23	-53	27	2390	0.55	0.00244
Posterior lobe	Right	Tuber	*	38	-54	-40	4338	-0.69	0.000048
Occipital	Left	Lingual gyrus	17	-14	-97	-27	2502	-0.65	0.0002

Abbreviations: BA = Brodmann Area; * = region not affiliated with a BA

[1] the MNI coordinates reported here are a direct conversion of the Talairach coordinates presented in the paper using the Yale BioImage Suite (1.0.0, 2018/12/12) online tool found at [https://bioimagesuiteweb.github.io/webapp/mni2tal.html].

Table 4. Coordinates of correlations between FFMQ scores and functional connectivity with the CEN.

			MNI Coordinates[1]						
Region	Hemisphere	Gyrus	BA	X	Y	Z	Cluster size	*r*	*p*
**Trait Mindfulness (FFMQ$_{Total}$)**									
Frontal	Right	Superior frontal	6	26	30	68	3490	-0.60	0.000805
**Observing**									
Occipital	Right	Lingual gyrus	19	29	-70	-2	2961	0.76	0.000002
Sub-lobar	Left	Lentiform nucleus	P	-23	10	-4	2872	0.64	0.000261
Posterior lobe	Right	Uvula	*	28	-77	-34	2256	0.63	0.00035
**Describing**									
Frontal	Left	Precentral gyrus	6	-32	-3	35	31308	0.65	0.000202
Parietal	Left	Precuneus	7	13	-66	39	17808	0.67	0.000109
Occipital	Right	Fusiform gyrus	19	25	-75	-20	18108	-0.58	0.00114
**Acting**									
No grey matter found			*	17	23	70	3548	-0.71	0.000026
Occipital	Right	Precuneus	31	2	-65	28	2315	-0.65	0.000159
Parietal	Right	Precuneus	19	37	-85	39	2262	-0.59	0.00107
**Non-reactivity**									
Temporal	Left	Superior temporal	41	-49	-29	-1	2229	-0.60	0.000718

Abbreviations: BA = Brodmann Area; P = putamen; * = region not affiliated with a BA

[1] the MNI coordinates reported here are a direct conversion of the Talairach coordinates presented in the paper using the Yale BioImage Suite (1.0.0, 2018/12/12) online tool found at [https://bioimagesuiteweb.github.io/webapp/mni2tal.html].

Table 5. Coordinates of correlations between FFMQ scores and functional connectivity with the ATN.

Region	Hemisphere	Gyrus	MNI Coordinates[1]						
			BA	X	Y	Z	Cluster size	*r*	*p*
**Observing**									
Frontal	Right	Middle frontal	9	36	13	27	5150	0.70	0.000035
Sub-lobar	Left	Insula	13	-43	2	21	3037	0.75	0.000004
Parietal	Left	Supramarginal	40	-66	-53	37	2141	-0.61	0.000592
**Describing**									
Temporal	Right	Superior temporal	22	50	-18	-13	1731	-0.67	0.000094
	Left	Superior temporal	22	-52	-45	9	1638	-0.68	0.000066
**Acting**									
Occipital	Right	Lingual gyrus	19	17	-57	-6	3256	-0.69	0.000051
**Non-judging**									
Frontal	Right	Superior frontal	9	12	60	31	1686	0.63	0.00031

Abbreviations: BA = Brodmann Area

[1] the MNI coordinates reported here are a direct conversion of the Talairach coordinates presented in the paper using the Yale BioImage Suite (1.0.0, 2018/12/12) online tool found at [https://bioimagesuiteweb.github.io/webapp/mni2tal.html].
